# Age, β-endorphin, and sex dependent effects of maternal separation on locomotor activity, anxiety-like behavior, and alcohol reward

**DOI:** 10.3389/fnbeh.2023.1155647

**Published:** 2023-04-05

**Authors:** Madison R. Scopano, Holly E. Jones, Sam G. Stea, Maya Z. Freeman, Judith E. Grisel

**Affiliations:** Department of Psychology and Neuroscience Program, Bucknell University, Lewisburg, PA, United States

**Keywords:** early life stress, opioid, EtOH reinforcement, conditioned place preference, sex differences, adolescent, development, adverse childhood experiences

## Abstract

**Introduction:**

Childhood adversity is pervasive and linked to numerous disadvantages in adulthood, including physical health problems, mental illness, and substance use disorders. Initial sensitivity to the rewarding effects of alcohol predicts the risk of developing an alcohol use disorder, and may be linked to developmental stress. The opioid peptide β-endorphin (β-E) regulates the stress response and is also implicated in the risk for excessive alcohol consumption.

**Methods:**

We explored the influence of β-E in an animal model of early life adversity using controlled maternal separation by evaluating changes in locomotor activity, anxiety-like behavior, and the initial rewarding effects of alcohol in a single exposure conditioned place preference paradigm in control C57BL/6J and β-E deficient β-E +/+ 0.129S2-Pomc tm1Low/J; β-E −/− mice. Maternal separation (MS) occurred for 3 h each day from post-natal days (PND) 5–18 in approximately half the subjects.

**Results:**

Maternal interactions increased following the separation protocol equally in both genotypes. MS and control subjects were tested as adolescents (PND 26–32) or adults (PND 58–72); the effects of MS were generally more pronounced in older subjects. Adults were more active than adolescents in the open field, and MS decreased activity in adolescent mice but increased it in adults. The increase in adult activity as a result of early life stress depended on both β-E and sex. β-E also influenced the effect of maternal separation on anxiety-like behavior in the Elevated Plus Maze. MS promoted rewarding effects of alcohol in male β-E deficient mice of either age, but had no effect in other groups.

**Discussion:**

Taken together, these results suggest that the effects of MS develop over time and are β-E and sex dependent and may aid understanding of how individual differences influence the impact of adverse childhood experiences.

## 1. Introduction

Adverse childhood experiences are common, potentially traumatic events that occur during childhood (Centers for Disease Control and Prevention, 2021). Early life stress is a major risk-factor for behavioral and health disorders in adults, including anxiety, PTSD, depression, and suicidality (Chang et al., 2019; Hadwen et al., 2022; McKay et al., 2022). For example, adverse childhood experiences predict the severity of substance use disorders (Dube et al., 2006; LeTendre and Reed, 2017; Grummitt et al., 2022) and a parental substance use disorder is a prevalent form of childhood adversity (van Draanen and Aneshensel, 2022) reflecting a transgenerational cycle of suffering. Childhood adversity also predicts more frequent and severe anxiety symptoms in adults (Hayward et al., 2020; King, 2021).

The effects of childhood adversity are age-dependent, in terms of the period of vulnerability as well as the onset of negative outcomes. For example, exposure to trauma between ages 3 and 5, a period of rapid brain and behavioral development, is associated with higher risk for PTSD (Kaplow and Widom, 2007; Schoedl et al., 2010) and also depression and suicidality (Turecki et al., 2014; Herzog and Schmahl, 2018) than exposure at other ages. Other studies in humans demonstrate a link between pre-pubertal childhood adversity and alcohol dependence in adolescence to early adulthood (Hill et al., 1994; Dube et al., 2002; Berent et al., 2018; Haugland et al., 2021). In fact, alcohol use disorders (AUDs) and anxiety disorders are exceedingly comorbid. Alcohol may be used to self-medicate anxiety disorders, which can lead to alcohol abuse (Gilpin et al., 2015; Logrip et al., 2017; Nentwig et al., 2019). In addition, alcohol-dependent patients are likely to suffer from anxiety as a result of neuroadaptation following chronic exposure to a sedative, and relapse is often precipitated by stress (Sinha, 2008; Lev-Ran et al., 2013; Gimeno et al., 2017). Initial sensitivity to alcohol reward is also linked with stress sensitivity and may be used to predict the risk of AUD (e.g., Ray et al., 2010, 2013).

Stress activates the hypothalamic-pituitary-adrenal (HPA) axis, a central stress response system (Chrousos, 2009). The hypothalamus releases corticotropin-releasing hormone (CRH) which has high affinity for the CRH1 receptor (CRH1R). CRH1R antagonists produce a reduction in binge-like drinking behavior (Koob, 2008). These effects are sex dependent. Male and female CRH1R knock-outs demonstrate reduced alcohol consumption but effects are more pronounced in females (Kaur et al., 2012) likely due to sex differences in CRH signaling (Bangasser et al., 2010). CRH can further stimulate the anterior pituitary to release adrenal corticotropic hormone (ACTH) and β-E. ACTH helps coordinate the peripheral stress response by signaling the adrenal cortex to release cortisol, while β-E provides negative feedback, inhibiting further release of CRH (Boyadjieva et al., 2009).

The opioid peptide β-endorphin (β-E) is integral to the relationship between stress and alcohol sensitivity (Nentwig and Grisel, 2019). Neuropeptides, like beta-endorphin, are believed to be implicated in AUD given their stress-buffering effects (Koob, 2008). β-E acts on mu-opioid receptors (Mansour et al., 1995) whereby the opioid system is well-known to modulate mood and alters stress physiology. Alcohol induces beta-endorphin release, promoting alcohol’s reinforcing properties (Olive et al., 2001; Contet et al., 2004; Jarjour et al., 2009). Moreover, low basal beta-endorphin levels are inversely correlated with alcohol abuse (Dai et al., 2005; Kiefer, 2006). Similar to CRH, effects of low beta-endorphin are sex dependent whereby β-E −/− males consume less EtOH, but β-E females more in comparison to control counterparts (McGonigle et al., 2016). β-E attenuates anxiety (Loh et al., 1976; Grisel et al., 2008). Thus, β-E deficiency may promote excess alcohol consumption to counteract a hyperactive HPA axis and contribute to the tendency to self-medicate with alcohol under stressful conditions (Aguirre et al., 1990; Gianoulakis, 1996; Nentwig and Grisel, 2019; Molina-Martínez and Juárez, 2021).

The literature in non-human animals clearly demonstrates that interruptions to maternal care have negative effects on the stress response to future adversity (Harlow and Zimmermann, 1959; Ladd et al., 1996; Huot et al., 2001; Daniels et al., 2004; Peña et al., 2017; Liu et al., 2018). Because the neurobiological mechanisms underlying the relationship between early adverse experiences, alcohol reward, and β-E are not well understood, we asked whether the effects of early stress on anxiety-like behaviors and alcohol reinforcement are influenced by β-E. To that end, we tested adolescent and adult, female and male, wild-type C57BL/6J and β-E-deficient mice to investigate how maternal separation affects anxiety-like behavior in open field and plus maze tests, as well as initial reward to alcohol in a single-exposure conditioned place preference (SE-CPP) assay. Our results suggest that the effects of maternal separation grow over time and depend upon both β-E and sex.

## 2. Materials and methods

### 2.1. Subjects

Experimentally naïve, male and female C57BL/6J (β-E +/+) and β-E deficient, B6.129S2-*Pomc^TM1Low^*/J (β-E −/− mice were used in all studies). β-E −/− mice were originally produced by standard techniques following the insertion of a premature stop codon into the *Pomc* gene (Rubinstein et al., 1996) and have since been fully backcrossed onto a C57BL/6J strain. β-E +/+ and β-E −/− mice for these studies were bred in-house from stock obtained from The Jackson Laboratories (Bar Harbor, ME) from same-sex parents. We bred 24 separate isogenic pairs to obviate the potential confound of RNA transmission (e.g., Liebers et al., 2014), and reduce the influence of litter effects, and checked for genetic differences in post-natal interactions (see below).

Mice were housed in standard Plexiglas cages with corn-cob bedding and a 3/4 in. cotton square for nesting material on Thoren racks with water and food available *ad libitum* in a temperature- (22 ± 2°C) and humidity-controlled (50 ± 20%) environment on a reverse 12:12 light:dark cycle with lights off at 09:30. Breeding pairs were checked each day, and litters were maintained with mother and father until weaning (PND-21). Half of the litters underwent separation from PND 5–18. After weaning, at PND 21, mice were housed 2–5/cage in standard Plexiglas caging on Thoren racks, by sex, in the same conditions as above, in a colony room adjacent to the breeding room. In total, 11 mating pairs were used in 13 crosses of the β-E +/+ C57BL/6J to obtain 88 pups, 37 control (CTL) and 51 maternal separation (MS); multiple litters from the same breeding pair were assigned to different experimental groups. In total, 14 crosses of the β-E −/− genotype, from 13 distinct breeding pairs, were made to obtain 91 pups, 37 CTL, and 54 MS. Male and female mice were included in all studies and tested as adolescents between PND 26 and 32 and/or adults from PND 58 to 74. We assessed 102 adolescents and 77 adults with *n* = 7–17 per age/treatment/genotype/sex. All procedures were in accordance with the National Institute of Health guidelines and approved by the Bucknell University Institutional Animal Care and Use Committee.

### 2.2. Open field apparatus

The open field apparatus consisted of a square arena (48 × 48 cm) enclosed by continuous, 40-cm-high walls made of black Plexiglas within a small (8’ × 8’) room used for this purpose. An overhead camera captured behavior for analysis by AnyMaze software (Stoelting, Wood Dale, IL, USA).

### 2.3. Elevated plus-maze apparatus

The elevated plus maze was made from Plexiglas and raised approximately 45 cm from ground level. The maze base was filled with wood shavings and enclosed by a clear 15 cm tall wall on its perimeter. The floor of the elevated portion of the maze was black. Two opposite arms (30 × 5 cm each) of the maze were enclosed by black, 10 cm high Plexiglas walls, and the remaining two arms were “open,” with a 2 mm wall. A center space (5 cm^2^) between these four arms was also not enclosed. An overhead camera captured behavior for analysis by AnyMaze software.

### 2.4. Conditioned place preference apparatus

The CPP apparatus has two distinct floors: one with circle-shaped tiles and the other with square-shaped tiles, both painted the same color red and enclosed by 30.5 cm high walls. The tile-textured sides (each 42 × 42 cm) were separated by a stimulus-neutral area with smooth white flooring (11.5 × 24 cm). The walls of the individual compartments and the whole apparatus were made of white, opaque Plexiglas. A 5 cm^2^ square hole is cut into the walls that separated the tile-textured contexts from the neutral area, and when inverted would allow the mouse to navigate throughout the whole apparatus on the test day (Grisel et al., 2014). An overhead camera captured behavior for analysis by AnyMaze software.

### 2.5. General experimental design

**FIGURE 1:**
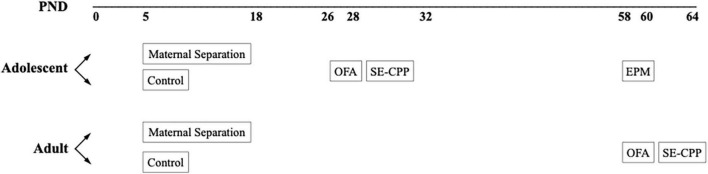
Schematic of the experimental design.

Figure 1 depicts the overall experimental design. Approximately half of the subjects were born into litters that were separated from their parents between PND 5–18, and the other half were undisturbed. Though this design cannot account for possible litter effects, systematic influences are less likely in a large breeding colony. Table 1 lists the number of subjects in each experimental group. Within each of these experimental groups, about half were assessed as adolescents and again as adults, and the other group was assessed only in adulthood. Testing times (adolescent and adult) were based on the literature (Stone and Quartermain, 1997; Spear, 2000; Varlinskaya et al., 2013). We made a concerted attempt to counterbalance testing across experimental groups (CTL vs. MS), sex, genotype (β-E +/+ vs. β-E −/−), and age (conditioning during adolescence vs. adulthood). All behavioral testing occurred 2–8 h into the animals active/dark cycle (1130–1730).

**TABLE 1:** The number of subjects in each of the 16 experimental groups.

Pup Tx	Strain	Sex	Test age	*N*
CTL	B6	Male	Adolescent	8
CTL	B6	Male	Adult	7
CTL	B6	Female	Adolescent	11
CTL	B6	Female	Adult	11
CTL	KO	Male	Adolescent	9
CTL	KO	Male	Adult	9
CTL	KO	Female	Adolescent	9
CTL	KO	Female	Adult	10
MS	B6	Male	Adolescent	16
MS	B6	Male	Adult	10
MS	B6	Female	Adolescent	16
MS	B6	Female	Adult	9
MS	KO	Male	Adolescent	17
MS	KO	Male	Adult	11
MS	KO	Female	Adolescent	17
MS	KO	Female	Adult	9

#### 2.5.1. Maternal separation

Separated litters were removed from their nest for a maternal separation protocol (Cruz et al., 2008), between PND 5–18, while others remained undisturbed in their home cage: Control (CTL) and Maternally Separated (MS). MS litters were placed in pre-warmed cages for 180 min/day from PND 5–18 between 12:00 and 13:00 ± 60 min. The pre-warmed cages contained 400 mL of corn-cob bedding and were kept atop a heating pad set between 60 and 65°C to maintain nest temperature between 32 and 34°C to prevent additional stress due to hypothermia. Ambient cage temperature measured after separation remained within ± 0.5°C of starting temperature. To encourage litter clustering, litters were placed on a clean, disposable coffee paper filter for the first 6 days of the separation protocol (PND 5–10). After separation, litters were placed back into home cages with their parents.

#### 2.5.2. Maternal interaction

Pre- and post-separation maternal care was assessed by trained undergraduates on PND-5, 8, 11, 14, and 17, based on published protocols (Own and Patel, 2013; de Almeida Magalhães et al., 2017). In separated litters, pre-separation maternal interactions were assessed for the 10 min immediately before the maternal separation period, and post-separation maternal interactions were assessed for 10 min immediately following separation. Control litters were assessed during approximately the same time periods, though litters were left undisturbed. During these periods, a dam was observed for either of two classes of activities every 30 s: maternal interaction or non-maternal interaction. Maternal interactions included nursing, licking/grooming, nest building, approaching pups, crouching over pups, and carrying pups. Non-maternal interactions included exploration of the environment, self-grooming, feeding, and resting. An interaction was marked if it persisted for at least 10 s during the 30 s observation period over the 10 min period.

#### 2.5.3. Open field activity (OFA)

Mice were assessed in the open field based on published protocols (Seibenhener and Wooten, 2015; Xu et al., 2021) either as adolescents (tested between PND 26 and 28) or adults (PND 58 and 68). Prior to the test, mice were brought in group cages from the colony room to a testing room across the hall where they habituated for 10–30 min. Each mouse was then placed individually in the center of the OFA apparatus and allowed free and uninterrupted movement for 15 min. The apparatus was cleaned with a low-residue detergent between each subject’s assessment. Locomotor activity was assessed by measuring distance traveled and average speeds. Anxiety-like behavior may be marked by decreased total locomotor activity (Sestakova et al., 2013) though interpretation of open field activity is not always straightforward (e.g., Crawley et al., 1997; Saré et al., 2021).

#### 2.5.4. Elevated plus maze (EPM)

Mice that were tested for OFA as adolescents were assessed for anxiety-related behavior in the EPM as adults (Walf and Frye, 2007; Grisel et al., 2008; Komada et al., 2008). Although this introduced an imbalance in the overall experimental design, we added this assessment in order to obtain an additional measure of anxiety-like behavior in this cohort and thought a repeat of the OFA test might be less informative due to habituation.

Following a 20 min habituation to the testing room in group cages, individual subjects were placed into the center area of the elevated maze with their head directed toward a closed arm, and allowed free and uninterrupted movement through the maze for 5 min. Following each individual test, the apparatus was cleaned with a sponge filled with dilute, low-residue detergent. The number of open arm entries, closed arm entries, and time spent in open arms were recorded for analysis. Open arm time (Walf and Frye, 2007) and the percentage of open arm entries (Rodgers and Dalvi, 1997) were calculated as measures of anxious behavior.

#### 2.5.5. Single-exposure conditioned-place preference

The single-exposure conditioned-place preference (SE-CPP) protocol was conducted over 5 days as described elsewhere (Grisel et al., 2014). Mice were subjected to the SE-CPP protocol between PND 28 and 35 or PND 60 and 75.

Each subject received a total of three injections, on three separate days: one of EtOH (1.5 g/kg; 20% vol:vol in physiological saline) and two of equivolume saline. Subjects were conditioned on days 1 and 3, left undisturbed days 2 and 4, and tested on day 5. The order of injection and the context-pairing were fully counterbalanced so that subjects received either 1.5 g/kg ethanol (EtOH) or equivolume saline and were immediately placed in either of two distinct contexts on day 1 for 30 min. On day 3, subjects that received EtOH on day 1 received saline, and those that received saline on day 1 were given EtOH on day 3. Day 3 conditioning took place in the alternate context, again for 30 min. On test day 5, the Plexiglas partitions between chambers were inverted, providing a hole that allowed the subjects access to both contexts from a neutral central chamber where they were placed immediately after saline injection. For this SE-CPP test, mice were allowed to roam around the apparatus for 30 min, during which time in each portion of the apparatus and the number of crossings was recorded. Following each individual test, the apparatus was cleaned with a sponge filled with dilute, low-residue detergent between each subject’s exposure. Percent time in the EtOH-paired context was calculated to measure alcohol reward (Grisel et al., 2014).

### 2.6. Statistical analysis

A 2-way (genotype and pup treatment), repeated measure ANOVA was used to assess maternal interactions in litters. For each behavioral measure that was tested in both adolescents and adults, we first analyzed the behavioral data using a 4-way Analysis of Variance (ANOVA; age at test, pup treatment, genotype, and sex). However, as our hypotheses were based on the effect of maternal separation and β-E, and because the plus maze assessment was only conducted in adults, we followed up using 3-way ANOVAs. For the assessment of EtOH place preference, we divided the time spent in the EtOH-paired context by the time spent in both contexts (subtracting out the time spent in the stimulus-neutral central portion of the apparatus). In addition to ANOVAs we also conducted one-sample, one-tailed t-tests to determine whether the percentage of time spent in an EtOH-paired context was greater than the null hypothesis of 50%. Data analysis was performed using SPSS 26.0. The criterion for significance in all cases was *p* ≤ 0.05.

## 3. Results

### 3.1. Maternal interactions

**FIGURE 2:**
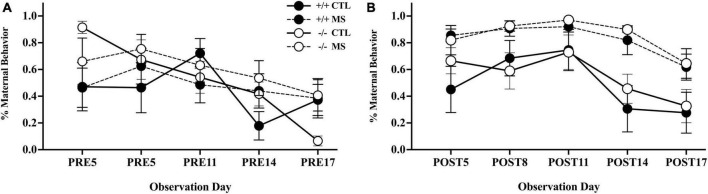
Maternal behavior pre- **(A)** and post- **(B)** separation, in control (CTL) or maternally separated (MS) litters of both genotypes. All litters were assessed twice over 10 mins on 5 days: PND 5, 8, 11, 14, and 17). The first assessments on each day are shown in panel **(A)** indicating no differences in pre-separation maternal behaviors between genotypes, or experimental groups. As expected, panel **(B)** shows an increase in maternal interactions in separated litters, an effect that did not differ between genotypes. Symbols represent the mean ± SEM percentage of time dams were engaged in maternal care in 30 litters (15 of each genotype). Please consult text for full experimental details and results.

We observed maternal behavior in the home-cage in maternally separated and control litters of β-E +/+ and β-E −/− animals, on PND-5, 8, 11, 14, and 17 (Figure 2). We analyzed maternal interactions using a 2-way (pup treatment and genotype) repeated measure ANOVA across the 10 observation periods, five pre- and five post-separation. There was no observable difference in the amount of maternal interactions between genotypes (F_1,24_ = 0.926, *p* > 0.05). Maternal separation led to more maternal interactions, as indicated by a main effect of treatment: F_1,24_ = 33.6, *p* < 0.01 (Figure 2B). This effect of separation on maternal interactions did not depend on genotype (F_1,24_ = 0.03, *p* > 0.05). There was a main effect of day with maternal attention decreasing over the course of development (F_1,24_ = 8.34, *p* < 0.01), but there was no interaction between day and genotype (F_1,24_ = 0.00, *p* > 0.05), day and pup treatment (F_1,24_ = 0.166, *p* > 0.05), nor triple interaction between day, genotype, and pup treatment (F_1,24_ = 0.03, *p* > 0.05).

### 3.2. Open field activity

**FIGURE 3:**
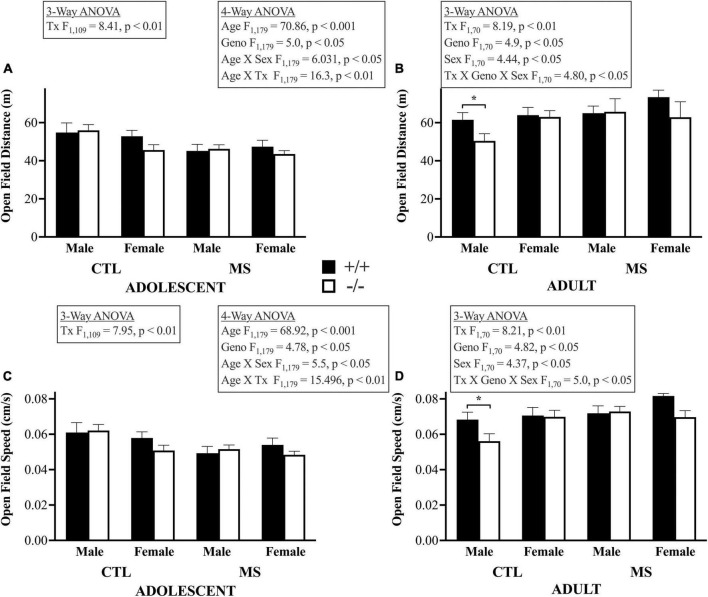
Locomotor distance and speed in the open field apparatus: bars depict means and SEM for Adolescent **(A,C)** and Adult **(B,D)** male and female, control (CTL) or maternally separated (MS) mice of both genotypes. Panels **(A,B)** show distance traveled in m during the 15 min test. The 4-way ANOVA demonstrated substantial changes in open field activity over time. These were dependent up sex and pup treatment, larger in female and maternally separated subjects. This was followed up by separate 3-way ANOVAs in each age group. In young mice, maternally separated mice were less active than controls. This pattern was also evident in adults, where there was also genotype (+/+ > −/−) and sex differences (F > M). Finally, in adults, a 3-way interaction demonstrated that the effect of maternal separation on distance traveled depended upon both sex and genotype. Panels **(C,D)** show a similar pattern with respect to the speed of locomotor activity in the Open Field Apparatus. There were main effects of Age, with adults quicker than adolescents. This was truer in females with β-E. Asterisks highlight significant *post hoc* differences. Group sizes ranged from 7 to 17, please see Table 1 for details. All significant group differences are highlighted in the textboxes. Please see the text for the complete results.

In the 4-way ANOVA, to examine the effects of age, genotype, pup treatment, and sex on distance traveled in the open field, we found a main effect of age, indicating that adults, tested around PND 60, traveled further than adolescents tested PND 26–28 (F_1,179_ = 70.86, *p* < 0.001). As shown in the top panels of Figure 3, there was no main effect of maternal separation on this measure of open field activity (F_1,179_ = 0.005, *p* > 0.05) or of sex (F_1,179_ = 0.321, *p* > 0.05). However, β-E deficient mice were less active than wildtype controls overall (F_1,179_ = 5.0, *p* < 0.05). Age differences depended on pup treatment (F_1,179_ = 16.31, *p* < 0.001) and sex (F_1,179_ = 6.03, *p* < 0.05) but there were no other significant interactions (Age X Geno, F_1,179_ = 0.884, *p* > 0.05; Tx X Geno, F_1,179_ = 0.16, *p* > 0.05; Tx X Sex F_1,179_ = 0.03, *p* > 0.05; Geno X Sex, F_1,179_ = 1.16, *p* > 0.05; Age X Tx X Geno, F_1,179_ = 0.007, *p* > 0.05; Age X Tx X Sex, F_1,179_ = 2.41, *p* > 0.05; Age X Geno X Sex, F_1,179_ = 0.796, *p* > 0.05; Tx X Geno X Sex, F_1,179_ = 1.73, *p* > 0.05, or Age X Tx X Geno X Sex, F_1,179_ = 3.34, *p* = 0.069). Because age had such a large influence on OFA, and to focus on our hypotheses, we followed up by separately analyzing adolescent and adult subjects in 3-way ANOVAs. In adolescents, maternal separation led to less locomotor activity (F_1,109_ = 8.85, *p* < 0.01). There were no influences of genotype, sex, nor significant interactions in younger mice. In adults, there was also a main effect of maternal separation (F_1,70_ = 8.2, *p* < 0.01), as well as of sex (F_1,70_ = 4.44, *p* < 0.05) and genotype (F_1,70_ = 4.9, *p* < 0.05) with females more active than males and β-E −/− mice less active than +/+ controls. Finally, in adults there was a significant triple interaction in adults between pup treatment, genotype, and sex (F_1,70_ = 4.8, *p* < 0.05). There were no other significant interactions (*p*’s > 0.05). The same pattern of results for locomotor speed in the Open Field assay were evident (Lower Panels, Figure 3). The 4-way ANOVA showed a main effect of age (F_1,179_ = 68.2, *p* < 0.001). There was no main effect of maternal separation on speed in the Open Field (F_1,179_ = 0.03, p > 0.05) or of sex (F_1,179_ = 0.427, *p* > 0.05). β-E deficient mice were less active than wildtype controls (F_1,179_ = 4.78, *p* < 0.05). Age differences depended on pup treatment (F_1,179_ = 15.5, *p* < 0.001) and sex (F_1,179_ = 5.5, *p* < 0.05). There were no other significant interactions: (Age X Geno, F_1,179_ = 0.928, *p* > 0.05; Tx X Geno, F_1,179_ = 0.095, *p* > 0.05; Tx X Sex F_1,179_ = 0.18, *p* > 0.05; Geno X Sex, F_1,179_ = 1.35, *p* > 0.05; Age X Tx X Geno, F_1,179_ = 0.003, *p* > 0.05; Age X Tx X Sex, F_1,179_ = 2.83, *p* > 0.05; Age X Geno X Sex, F_1,179_ = 0.936, *p* > 0.05; Tx X Geno X Sex, F_1,179_ = 2.50, *p* > 0.05, or Age X Tx X Geno X Sex, F_1,179_ = 2.64, *p* > 0.05). Again, we followed up by separately analyzing adolescent and adult subjects in 3-way ANOVAs. In adolescents, maternal separation led to slower locomotor activity (F_1,109_ = 7.95, *p* < 0.01). There were no influences of genotype or sex or significant interactions in adolescents. In adults there was also a main effect of pup treatment (F_1,70_ = 8.2, *p* < 0.01), as well as of sex (F_1,70_ = 4.37, *p* < 0.05) and genotype (F_1,70_ = 4.82, *p* < 0.05). Finally, there was a significant triple interaction in adults between pup treatment, genotype and sex (F_1,70_ = 5.0, *p* < 0.05). There were no other significant interactions (*p*’s > 0.05).

### 3.3. Elevated plus maze

**FIGURE 4:**
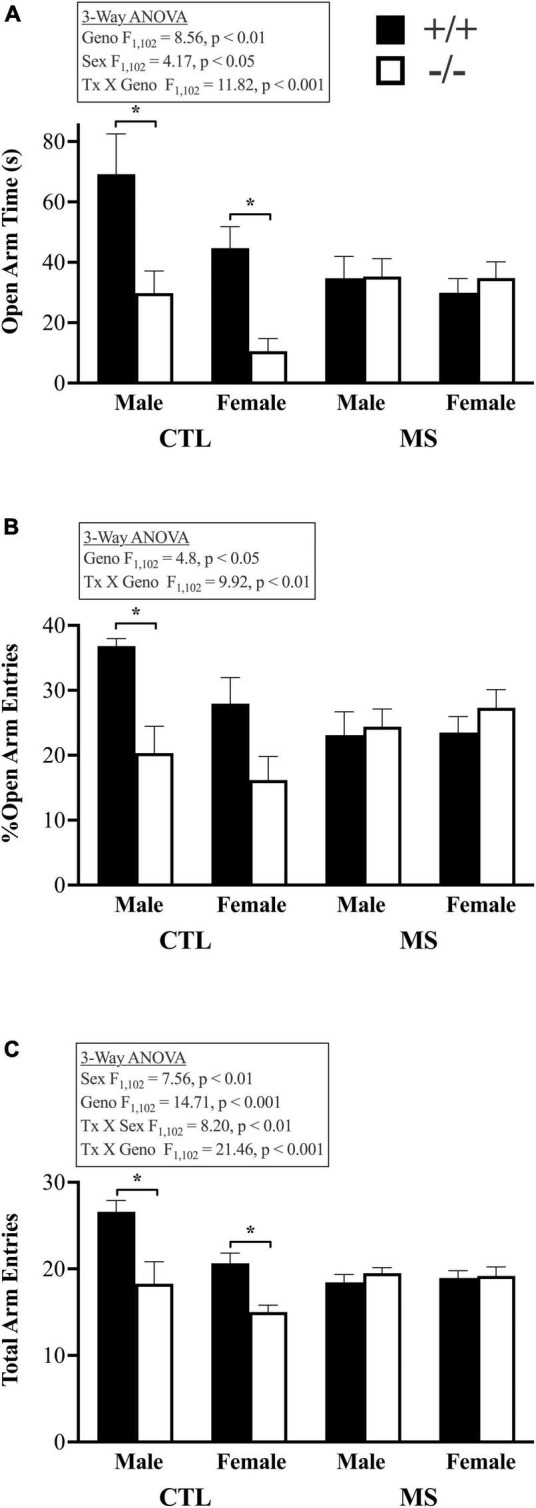
Behavior assessed in the elevated plus maze in adult, male and female, wildtype (+/+) and β-E deficient mice (−/−) either raised normally or maternally separated between PND 5–18. **(A)** Shows more anxiety-like behavior (reduced time spent in the open arms of the maze) in β-E deficient mice than controls when raised in standard conditions, but increased anxiety, leading to a loss of group differences, in wildtype mice following maternal separation. This pattern was also evident in the percentage of entries into open arms **(B)**. However, normally raised β-E deficient mice were less active than controls as shown in panel **(C)**. Bars represent group means and SEM; the number of subjects per group ranged from 7 to 11, please see Table 1 for details. All significant group differences are highlighted in the associated text and described fully in the results; asterisks highlight significant *post hoc* differences.

Adult mice that were tested in the OFA as adolescents were assessed in the EPM as adults (Figure 4). There were main effects of genotype, as well as significant interactions between pup treatment and genotype in all three measures of plus maze behavior. Mice lacking β-E spent less time in the open arms of the maze (Figure 4A; F_1,102_ = 8.57, *p* < 0.01). Females were also less likely to be in open arms than males (F_1,102_ = 4.1, *p* < 0.05). While there was no significant main effect of pup treatment (F_1,102_ = 1.36, *p* > 0.05) or interaction between pup treatment and sex (F_1,102_ = 2.39, *p* > 0.05), the two genotypes were differentially affected by maternal separation, as indicated by a significant treatment X genotype interaction (F_1,102_ = 11.82, *p* < 0.001). There were no other significant interactions with respect to the time spent in the open arms of the elevated plus maze. This pattern of results was similar in the percentage of open arm entries, calculated by dividing these by the total number of arm entries. Figure 4B shows main effects of genotype (F_1,102_ = 4.8, *p* < 0.05) but not pup treatment (F_1,102_ = 0.08, *p* > 0.05) or sex (F_1,102_ = 0.84, *p* > 0.05). There was a significant interaction between genotype and pup treatment (F_1,102_ = 9.99, *p* < 0.01) but no other significant interactions. Like in the OFA, β-E deficient mice were generally less active as evidenced by a main effect of genotype on the total number of arm entries (Figures 3, 4C; F_1,102_ = 14.71, *p* > 0.001). Females were less active than males overall (F_1,102_ = 7.56, *p* < 0.01) but there was no main effect of pup treatment (F_1,102_ = 1.83, *p* > 0.05). There was also a significant interaction between pup treatment and genotype (F_1,102_ = 21.45, *p* < 0.001) and pup treatment and sex (F_1,102_ = 8.25, *p* < 0.01) but no other significant interactions.

### 3.4. Single-exposure conditioned place preference (SE-CPP)

**FIGURE 5:**
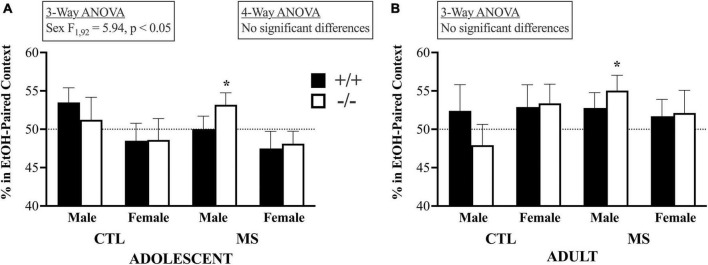
Initial subjective reward to EtOH as measured by a single-exposure conditioned place preference assay did not differ by age or treatment. In fact, there was no evidence of initial subjective rewarding effects of alcohol except in MS male β-E deficient mice that preferred a context previously associated with a single injection to 1.5 g/kg EtOH over one associated with saline injection.Panels **(A)** and **(B)** show choice behavior in adolescent and adult subjects, respectively. Bars depict group means and SEM, there were between 7 and 17 subjects in each group, and * indicates group means significantly above the null hypothesis of 50%. See text for full discussion of results.

A 4-way ANOVA of choice behavior on the SE-CPP test day, shown in Figure 5, revealed no significant main or interactive effects: Age, F_1,159_ = 3.214, *p* = 0.075; Pup Treatment, F_1,159_ = 0.032, *p* > 0.05; Genotype, F_1,159_ = 0.00, *p* > 0.05 or Sex, F_1,159_ = 1.84, *p* > 0.05; Age X Pup Treatment, F_1,159_ = 0.63, *p* > 0.05; Age X Geno, F_1,159_ = 0.10, *p* > 0.05; Age X Sex F_1,159_ = 2.91, *p* = 0.09; Tx X Geno, F_1,159_ = 1.70, *p* > 0.05; Tx X Sex F_1,159_ = 0.97, *p* > 0.05; Geno X Sex, F_1,159_ = 0.10, *p* > 0.05; Age X Tx X Geno, F_1,159_ = 0.008, *p* > 0.05; Age X Tx X Sex, F_1,159_ = 0.961, *p* > 0.05; Age X Geno X Sex, F_1,159_ = 0.126, *p* > 0.05; Tx X Geno X Sex, F_1,159_ = 1.44, *p* > 0.05, or Age X Tx X Geno X Sex, F_1,159_ = 0.046, *p* > 0.05. In adolescents, the 3-way ANOVA showed no influence of pup treatment (F_1,92_ = 0.241, *p* > 0.05) or genotype (F_1,92_ = 0.066, *p* > 0.05) on SE-CPP, but females spent a lower percentage of their time in the EtOH-paired context than males (F_1,92_ = 5.94, *p* < 0.05). Adolescent mice also evidenced no significant interactions between pup treatment and genotype (F_1,92_ = 0.932, *p* > 0.05), treatment and sex (F_1,92_ = 0.000, *p* > 0.05), or genotype and sex (F_1,92_ = 0.001, *p* > 0.05). There was also no triple interaction between pup treatment, genotype, and sex (F_1,92_ = 0.614, *p* > 0.05). The results were similar in adults: the 3-way ANOVA showed no significant influences on SE-CPP by pup treatment (F_1,67_ = 0.371, *p* > 0.05), genotype (F_1,67_ = 0.037, *p* > 0.05). Unlike adolescents, in adults there were no sex differences (F_1,67_ = 0.05, *p* < 0.05). There were no significant interactions between pup treatment and genotype (F_1,67_ = 0.75, *p* > 0.05), treatment and sex (F_1,67_ = 1.5, *p* > 0.05), or genotype and sex (F_1,67_ = 0.176, *p* > 0.05), nor was there a triple interaction between pup treatment, genotype, and sex (F_1,67_ = 0.780, *p* > 0.05). As described previously (Grisel et al., 2014) our primary interest in the analysis of SE-CPP is whether any group spends more than 50% of their time on day 5 in the context previously associated with EtOH. Toward that end, we analyzed each group separately by one-sample, one-tailed, *t*-tests. Maternal separation led to SE-CPP only in male, β-E-deficient mice (of either age): In adolescents: male β-E +/+ control (CTL), t_7_ = 1.8, *p* = 0.057; male β-E −/− CTL, t_8_ = 0.42, *p* = 0.343; female β-E +/+ CTL, t_10_ = −0.64, *p* = 0.269; female β-E −/− CTL, t_5_ = −0.499, *p* = 0.320; male β-E +/+ maternally separated (MS), t_12_ = 0.013, *p* = 0.495; male β-E −/− MS, t_18_ = 2.04, *p* = 0.028; female β-E +/+ MS, t_13_ = −1.12, *p* = 0.141; female β-E −/− MS, t_11_ = - 0.152, *p* = 0.137. In adults the pattern of results was the same: male β-E +/+ control (CTL), t_5_ = 0.645, *p* = 0.274; male β-E −/− CTL, t_8_ = −0.754, *p* = 0.236; female β-E +/+ CTL, t_10_ = 0.988, *p* = 0.173; female β-E −/− CTL, t_9_ = 1.367, *p* = 0.102; male β-E +/+ maternally separated (MS), t_9_ = 1.369, *p* = 0.102; male β-E −/− MS, t_5_ = 2.52, *p* = 0.027; female β-E +/+ MS, t_7_ = 0.739, *p* = 0.242; female β-E −/− MS, t_6_ = 0.715, *p* = 0.251.

## 4. Discussion

Consequences of stress early in life are far reaching, affecting a wide range of psychological and physical outcomes in adults (Felitti et al., 1998; Hughes et al., 2017, for example). In order to better understand how early stress influences adult functioning, we investigated contributions of the endogenous opioid β-E to the effects of maternal separation on locomotor and anxiety-like behavior, as well as the initial rewarding effects of alcohol in mice. Following maternal separation, we evaluated wildtype and β-E deficient mice as adolescents or adults in the open field assay (OFA), elevated plus maze (EPM; adults only), and alcohol single-exposure conditioned place preference assay (SE-CPP). We found that effects of maternal separation are more pronounced in adults than in adolescents, and depend on β-E and sex.

In rodents, maternal separation (MS) between 180 and 360 min disrupts maternal care and is associated with a variety of negative outcomes in adulthood (for example, see: Huot et al., 2001; Pryce and Feldon, 2003; Rüedi-Bettschen et al., 2006; Cruz et al., 2008; Amini-Khoei et al., 2019; Bian et al., 2021). However, variation in separation parameters, timing and type of dependent measures, and strains and species employed, contribute to substantial variability in the basic science literature (Nylander and Roman, 2013; Tractenberg et al., 2016; Wang et al., 2020). In our hands, 3 h of separation on post-natal days 5–18 led to more frequent maternal interactions upon reunion (Millstein and Holmes, 2007). The increase in maternal attention was not influenced by β-E, suggesting that the genotypic differences in adolescent and adult behavior we observed are not due to differential parental care, but rather to direct effects of the peptide, or to its interactions with the separation protocol.

We studied activity in a novel open field in both adolescents and adults. Maternal separation (MS) reduced locomotion during adolescence, but increased it in adults, showing that the effect of early interventions varies across the lifespan. These influences were fairly subtle, and in adults, depended on an interaction between sex and β-E. That is, there was a sexually dimorphic influence of β-E, a pattern we have seen before (Barfield et al., 2010; Rhinehart et al., 2018, 2020; Nentwig et al., 2019). Here, MS +/+ females, and −/− males, were more active as adults than normally reared or opposite sex counterparts. Although it is possible that increased activity reflects a more anxious state (Zimprich et al., 2014). Our previous findings, including measurements of cortisol and *Crh* expression (Nentwig et al., 2019) suggest that reduced activity may be an indication of increased anxiety as others have suggested (for example, Sestakova et al., 2013). Future studies should evaluate activity in different regions of the open field, or include additional measures of anxiety like behavior to corroborate this hypothesis.

We found that the effect of MS on OFA develops over time, and is influenced by β-E in a sex-dependent manner. Others have found sex-specific effects of MS (Eklund and Arborelius, 2006; Bondar et al., 2018; Gildawie et al., 2021; Knox et al., 2021, for example), though until recently, many basic neuroscience studies have not included sex as a biological variable (Zucker et al., 2022). Perhaps for this reason, studies on the role of β-E in MS that include males and females are scarce, or absent. Nonetheless, a seminal article in the area by D’Amore et al. (1993) found that male CD-1 mice, subjected to handling stress from PND 2–19, had increased β-E tone in adolescence relative to non-handled counterparts. This pattern was apparently reversed by PND 50, as handled subjects were substantially less responsive to intracerebroventricular β-E injection as measured by tailflick latency. Whether a stress-induced increase in young mice leads to tolerant older ones, as the authors suppose, and the neural mechanisms by which β-E contributes to antinociception and locomotion overlap, there seems to be clear evidence of developmentally driven alterations β-E that contribute to behavioral changes. Strain differences in the effect of MS have also been demonstrated (Millstein and Holmes, 2007; Tractenberg et al., 2016) suggesting that specific neural and endocrine variation (Charney, 2004) modulate effects of early stress. Our data support the notion that the balance between resilience and fragility across development is influenced by β-E signaling.

In the two locomotor assessments that we evaluated in both adolescents and adults–OFA distance and speed–MS had opposite effects in younger and older mice. Though most studies focus on the effects of early life stress only in adults (Romeo et al., 2003; Parfitt et al., 2004; Jacobson-Pick and Richter-Levin, 2010; Malcon et al., 2020; Kestering-Ferreira et al., 2021), Marin and Planeta (2004) found increased locomotor responses to a novel environment as well as cocaine in adolescents, but not adults, following 5 h MS from PND 2 to 6. Others have noted developmental changes in the effects of stress suggesting reduced resilience in adults compared to adolescents, such as a study by Meyer et al. (2021). On the other hand, He et al. (2020) subjected male and female C57BL/6 mice to maternal separation for 4 h a day, PND 2–12, and assessed anxiety-like behaviors during adolescence and again in adulthood. There was no effect of MS in mice repeatedly tested in OFA and EPM, a day apart on PND 30–31, PND 41–42, and PND 75–76, though a single assessment of immobility in the forced swim test on PND 51 showed more immobility in both male and female MS groups. Because this study involved repeated use of the same tests, insensitive adolescents may have habituated to the protocol leading to a lack of effect in adults.

The alterations seen in the distance and speed of locomotion in our study as a function of pup treatment may reflect effects of early manipulation on dopamine signaling or circuitry related to anxiety or both, and future studies will be needed to elucidate underlying mechanisms. In terms of anxiety-like behavior, rather than expose subjects to the open field twice, first around 4 weeks and then again around 9 weeks, we tested those that had been assessed in the open field as adolescents in the elevated plus maze as adults. Again, there was a treatment and genotype interaction in that the influence of maternal separation on anxiety-like behavior in the EPM depended on β-E. Wildtype C57BL/6J mice that had undergone the neonatal MS protocol spent less time, and had fewer entries, in the open arms of a plus maze than their normally reared counterparts. However, because β-E deficient mice are more anxious to begin with (Loh et al., 1976; Grisel et al., 2008) there may have been a floor effect in these subjects. Future studies might shed light on factors influencing the relationship between MS and anxiety by evaluating EPM behavior in multiple age groups, and/or employing different assays of anxiety such as the Light-Dark box. However, our data support the hypothesis that younger animals are more resilient to the effects of early stress, which becomes more likely with increasing age. In contrast to OF activity, there was evidence of reduced overall activity in MS +/+ adults tested in the plus maze, perhaps reflecting higher stakes of exploration on a narrow, elevated platform.

The single-exposure conditioned place preference assay (SE-CPP) is designed to probe sensitivity to rewarding effects of the first drug exposure (Grisel et al., 2014; Nentwig et al., 2017). In this paradigm there is no habituation to the apparatus. The protocol is unbiased, and fully counterbalanced so that half of the subjects get EtOH on the first conditioning day and saline on the second, and the other half have their injections in the opposite order. Injections are associated with either of two distinct contexts. In addition to looking for group differences, we assess SE-CPP by determining whether any group elects to spend more than half their time in the EtOH-paired context on the test day. We have seen SE-CPP in multiple strains of mice, as well as Sprague Dawley rats, yet there was little evidence in the present study for place preference aside from maternally separated β-E deficient males. Though the evidence for SE-CPP was modest in these two cohorts, the fact that it was evident in both adolescent and adult subjects makes it more likely that this group is particularly sensitive to EtOH reward upon the first exposure.

The endogenous opioid peptide, β-E, has been implicated in EtOH consumption in humans (cf: Herz, 1997). In the clinic, basal levels of plasma β-E, as well as a rise in this peptide following alcohol administration, correlate with a heritable risk for high drinking (Dai et al., 2005; Kiefer et al., 2006) and a *POMC* haplotype marker has been associated with alcoholism in women, but not men (Racz et al., 2008). However, the mechanisms responsible for these influences remain unknown. β-E deficiency is thought to promote excess alcohol consumption to counteract a hyperactive HPA axis (Aguirre et al., 1990; Gianoulakis, 1996; Nentwig and Grisel, 2019; Molina-Martínez and Juárez, 2021) and given higher baseline anxiety in β-E deficient mice on the EPM, we might expect greater SE-CPP in these groups to be exacerbated by MS, as early life stress increases reward insensitivity (Weller and Fisher, 2013; Hanson et al., 2017). EtOH-reinforcement may be positive, that is, based on pleasurable effects of the drug, negative, due to its ability to reduce aversive states, or a mix of both. Because habituation to injections or the testing apparatus diminishes SE-CPP (Grisel et al., 2014), we hypothesize that negative reinforcing effects of EtOH predominate in this paradigm. Unpublished results from our laboratory support that contention: we found a tendency for higher anxiety-like behavior (assessed in the EPM and Light-Dark Box) to predict higher SE-CPP in Swiss Webster mice. While we lacked sufficient power to test this relationship in the present study, the correlation between the percentage of open arm entries and the percentage of time spent in the EtOH-paired context was highest in β-E deficient male mice that had been maternally separated (though not significant, 2-tailed *p* = 0.089). We are presently testing this hypothesis in a larger sample of diversity outbred mice, again by looking for phenotypic correlations between anxiety-like behavior and SE-CPP, but as that study is incomplete, we can only speculate that enhanced sensitivity to stress in MS, β-E deficient, mice might override habituation to experimenters and other aspects of the experimental protocol involving repeated testing, to promote alcohol reward.

One limitation of the present study involves the possibility of litter effects, since littermates are more likely to be similar, and in our case the post-natal separation was applied to whole litters. Despite the large number of subjects in this study, our group sizes, usually two to three subjects per strain, sex and treatment from the same parental lineage, do not provide sufficient power for a mixed-effects model (Lazic and Essioux, 2013). However, the large number of breeding pairs used in this study, counterbalanced group assignments from single breeding pairs, reduce the likelihood of Type I errors due to within-litter similarity of offspring.

Although we have not previously found sex differences using the SE-CPP model, there is ample evidence that stress exposure affects males and females differently, and we have also seen that this relationship is especially evident in β-E deficient mice. Because β-E has been shown to moderate stress by inhibiting CRH, a lack produces a hyper-stress state (Grisel et al., 2008; McGonigle et al., 2016; Nentwig et al., 2019). Sillaber et al. (2002) investigated the effects of repeated stress on drinking in mice lacking the CRH receptor, another animal model of increased stress susceptibility. Effects of repeated forced swim were not evident at first, but began to emerge around 3 weeks after the stress protocol, and remained evident for several months. This study only included adult males, but nonetheless suggests that consequences of stress incubate to become more evident over time. Our study took place across less than 10 weeks, and it may be that an extended period, such as the 9-month protocol employed by Sillaber and colleagues, would unmask bigger effects of MS. The Sillaber study evaluated oral self-administration, which may have different underpinnings that the SE-CPP we evaluated.

## 5. Conclusion

Early life stress is a major risk-factor for behavioral and health disorders. This broad field of research indicates that the effects of stress during periods of rapid brain development are multifarious and persistent. In part because the nature of stress is itself so elusive and complex, the mechanisms underlying the associations between early adverse experiences and adult outcomes are ripe for investigation. The current study adds to a rapidly expanding literature to suggest that opioid signaling contributes to the influence of early adverse experiences on adult outcomes. In particular, we have shown that β-E, long known to be involved in regulating the neuroendocrine stress response, impacts the effect of MS on exploration of a novel field, alters anxiety-like behavior, and may contribute to sex differences in sensitivity to alcohol reward. Taken together, these findings suggest that lasting impacts of early adversity involve complex genetic, neuroendocrine and environmental underpinnings.

## Data availability statement

The raw data supporting the conclusions of this article will be made available by the authors, without undue reservation.

## Ethics statement

The animal study was reviewed and approved by Bucknell University Institutional Animal Care and Use Committee.

## Author contributions

MS performed data acquisition. HJ assisted with EPM and SE-CPP data collection. SS and MF assisted with MS data acquisition. MS and JG designed the study, performed data analysis, and wrote the manuscript. All authors contributed to the article and approved the submitted version.
